# Characterisation of Engineered Nanomaterials in Nano-Enabled Products Exhibiting Priority Environmental Exposure

**DOI:** 10.3390/molecules26051370

**Published:** 2021-03-04

**Authors:** Raisibe Florence Lehutso, Yolanda Tancu, Arjun Maity, Melusi Thwala

**Affiliations:** 1 Water Centre, Council for Scientific and Industrial Research, Pretoria 0001, South Africa; FLehutso@csir.co.za (R.F.L.); ytancu@csir.co.za (Y.T.); 2 Department of Chemical Sciences, University of Johannesburg, Johannesburg 2006, South Africa; AMaity@csir.co.za; 3 DST/CSIR, Centre for Nanostructure and Advanced Materials (CeNAM), Council for Scientific and Industrial Research, Pretoria 0001, South Africa; 4 Department of Environmental Health, Nelson Mandela University, Port Elizabeth 6031, South Africa

**Keywords:** nano-enabled products, engineered nanomaterials, exposure potential, characterisation

## Abstract

Analytical limitations have constrained the determination of nanopollution character from real-world sources such as nano-enabled products (NEPs), thus hindering the development of environmental safety guidelines for engineered nanomaterials (ENMs). This study examined the properties of ENMs in 18 commercial products: sunscreens, personal care products, clothing, and paints—products exhibiting medium to a high potential for environmental nanopollution. It was found that 17 of the products contained ENMs; 9, 3, 3, and 2 were incorporated with nTiO_2_, nAg, binaries of nZnO + nTiO_2_, and nTiO_2_ + nAg, respectively. Commonly, the nTiO_2_ were elongated or angular, whereas nAg and nZnO were near-spherical and angular in morphology, respectively. The size ranges (width × length) were 7–48 × 14–200, 34–35 × 37–38, and 18–28 nm for nTiO_2_, nZnO, and nAg respectively. All ENMs were negatively charged. The total concentration of Ti, Zn, and Ag in the NEPs were 2.3 × 10^−4^–4.3%, 3.4–4.3%, and 1.0 × 10^−4^–11.3 × 10^−3^%, respectively. The study determined some key ENM characteristics required for environmental risk assessment; however, challenges persist regarding the accurate determination of the concentration in NEPs. Overall, the study confirmed NEPs as actual sources of nanopollution; hence, scenario-specific efforts are recommended to quantify their loads into water resources.

## 1. Introduction

Engineered nanomaterials (ENMs) are a case of emerging contaminants and can be emitted from nano-enabled products (NEPs) into water systems. Hence, it is essential to establish the physico-chemical properties of ENMs incorporated in NEPs in order to appropriately examine the environmental exposure potential and implications that may arise across the lifecycle of the products [1]. While the reporting on NEPs’ market penetration dates back to 2004 [2,3], the identities and physico-chemical properties of the incorporated ENMs are not well known as they are not commonly reported by manufacturers or the information is very minimal [4,5]. For example, in the Woodrow Wilson International Centre for Scholars and the Project on Emerging Nanotechnology Inventory [6], the incorporated ENMs’ types and respective physico-chemical properties are unknown in 49 to 60% of the listed NEPs [2,5,7].

In the pursuit of addressing the health, safety, and environmental concerns linked to ENMs, it is paramount to gain insights into the properties of ENMs across potential nanopollution sources, including NEPs. However, the relatively minimal efforts of this nature have experienced analytical challenges due to inadequate and non-standardised techniques to characterise ENMs in complex matrices of NEPs [8]. For instance, a technique such as single-particle inductively coupled plasma mass spectrometry (spICP-MS) has only been recently developed for the detection and size characterisation of metal-based ENMs in environmental samples [9,10,11], but it may still not be the best fit to quantify and characterise ENMs in NEPs [12,13], for instance, if sample preparation is not tailored efficiently. Similarly, the commonly applied dynamic light scattering technique for the hydrodynamic size determination of ENMs in suspension also has its limitations [14,15] (e.g., prone to influence by larger size particles). Lastly, some of the relatively efficient techniques are not common pieces of equipment in general environmental laboratories.

Nonetheless, advances have been made in this niche of research despite the persisting analytical challenges, resulting in better knowledge and understanding of the characteristics of ENMs in NEPs [16,17,18,19,20,21,22,23,24,25,26,27]. Overall, the literature indicates that the ENMs type, quantities, and characteristics in NEPs tend to vary widely even for closely related product types. For example, the commonly incorporated nTiO_2_ in sunscreens vary in morphology (elongated, angular, irregular), size (18 to 90 nm), surface area (9.1 to 60.8 m^2^/g), and phase (rutile, anatase, or both) properties [17,18,19,28,29,30]. Hence, it is not simple to make solid assumptions and estimations based on a few studies, but rich datasets need to be developed in order to strengthen modeling exercises used to predict the environmental fate and effects of ENMs released from NEPs.

The NEPs’ environmental exposure potential for ENMs (likelihood to nanopollute) is a critical determinant factor for the environmental fate and effects of ENMs [31]. However, the varying factors such as release potential on ENMs suspended in liquids, surface-bound, solid, or nanostructured influences the exposure potential. [5,31]. Aquatic environments are most likely to experience medium to high exposure extent from NEPs whose ENMs are suspended in liquids, followed by surface-bound [18,32]. Different global inventories [5,18] indicate that NEPs categorised as having medium to high release potential to aquatic environments are common in markets and as much as 36–96% of the NEPs are being sold. These NEPs are in the product categories of health and fitness (e.g., personal care products, sunscreens, cosmetics) as well as home and garden (cleaning products, paints, laundry cleaning products) [5,7,18]. From the different NEPs categories, sunscreens are the most widely studied [17,18,19,20,21,23,24,25,26], despite the evidence of high ENMs’ environmental exposure potential in NEPs such as paints [33,34,35,36,37] and other personal care products/cosmetics (e.g., toothpaste, shampoos, and face creams) [38,39]. While nTiO_2_ can be expected to be emitted at relatively higher volumes due to their wide application in a spectrum of NEPs, the incorporation of other ENM types such as nAg, nSiO_2_, and nZnO have been reported in other market active NEPs within the health and fitness, as well as home and garden categories [7,18,32]. Due to various facets of data required, other key environmental exposure determinants, for instance, market parameters and life cycle dynamics, tend to be more complex to examine.

The data for ENMs’ properties are not readily suitable for direct cross-application for environmental exposure and toxicity assessments even within and across NEPs categories [40,41,42]; hence, the data generation needs expansion to other types of top-selling commercial NEPs, especially those exhibiting medium to high likelihood for the release of ENMs (i.e., nanopollution). As such, the current study aimed to determine the characteristics of the ENMs for a wide array of commercial NEPs product categories. The studied products were manufacturer-labelled to be NEPs and considered to exhibit medium to high environmental exposure potential for ENMs for water systems [18,32]. Additionally, products suspected to be incorporated with ENMs, either based on product description, or due to similarity to previously identified NEPs, but still meeting the criteria of medium to high environmental exposure, were also included in the study samples. The “suspect” products were included to demonstrate that NEPs may extend wider than the products declared by manufacturers, and hence potentially a greater nanopollution extent than currently estimated. The “suspect products” have previously not attracted attention in ENMs exposure assessments mainly because of the lack of a nano declaration or labelling by manufacturers. The study focused on some top-priority ENMs’ parameters for risk characterisation data requirements [43], specifically the particle size distribution, shape, phase, surface charge, composition, and concentration. Overall, the study was exclusive to top priority NEPs’ potential sources for nanopollution in water systems.

## 2. Materials and Methods

### 2.1. Selection of NEPs

Eighteen products from the health and fitness, home and garden product categories were purchased from South African retailers, as shown in Table 1.

#### 2.1.1. Characterisation of ENMs

The physico-chemical characteristics of ENMs obtained from the NEPs were determined using scanning electron microscopy coupled to energy-dispersive X-ray (SEM-EDX), high-resolution transmission electron microscopy coupled with energy-dispersive (TEM-EDX), dynamic light scattering (DLS), and X-ray powder diffraction (XRD).

The particle shape, size, and elemental composition were determined using SEM-EDX and TEM-EDX. For SEM-EDX (ZEISS Supra55, Oberkochen, Germany) analysis, a small amount of the sample was deposited onto the double-sided carbon tape, which was mounted on a copper stub. The samples were sputter-coated with carbon using a Turbo carbon evaporator (EMITECH–K950X, London, United Kingdom) to prevent charging during SEM-EDX analysis. For TEM-EDX (JEOL-JEM 2100, Tokyo, Japan), nTiO_2,_ nSiO_2_, nZnO and nAg-based samples were respectively dispersed in ethanol (95%, Merck, Johannesburg, South Africa) and Milli-Q water (18 MΩ.cm). The samples were sonicated in a water bath (Ultrasonic bath RK 514 BH cap, Labotec, Johannesburg, South Africa) for 5 min. The Cu grid with a holey carbon film was dipped into the sample solution multiple times and air-dried for 12 h. Image J software was used to calculate the size of the particles.

The ENMs’ zeta potential was determined using DLS (Malvern Zetasizer Nano ZS, Worcestershire, United Kingdom). First, 5 mg of the sample extract (SUN1–5, LB1, CA1–2, CM1, PA1–5, SK1) was dispersed in 10 mL of Milli-Q water and sonicated for 5 min in a sonication bath at 25 °C. SAN1 and SAN2 were analysed as purchased. All suspensions were filtered using a 0.45 µm syringe filter (Merck, Johannesburg, South Africa) and analysed for zeta potential at 25 °C.

The ENMs’ phase was determined using XRD (PANalytical XPERT-PRO diffractometer, Malvern, United Kingdom). Briefly, the samples were deposited on a sample holder and analysed using the XRD diffractometer equipped with Cu Kα radiation (λ = 1.540598 Å), with a variable slit at 45 kV and 40 mA. The sample was scanned with a 2Ɵ range of 5–90°. The International Centre for Diffraction Data (ICDD) database was used to reference the XRD patterns.

#### 2.1.2. Pre-Treatment of NEPs’ Samples

Due to the complex matrix of the NEPs, most samples (except for SAN1 and SAN2) could not be analysed directly (as purchased) and required some sample pre-treatment. Different ENMs isolation/extraction methods were used depending on the NEPs’ matrix. Following ENMs isolation, the samples were prepared for characterisation using the techniques mentioned in Section 2.1.1. Since SAN1–2 required no sample pre-treatment, the ENMs in the NEPs suspension were directly examined.

##### Sample Pre-Treatment for SUN1–5, LB1

Pre-treatment of the NEPs’ samples matrix was performed by following previously developed methods [1,2,3]. Briefly, 2 g of the product was extracted with 30 mL of methanol, hexane, chloroform, and 1% Triton (pH12) (1:1:1:1 ratio). The suspension was vortexed for 5 min, sonicated for 30 min at 25 °C, and centrifuged at 10,000 rpm for 15 min. The supernatants were discarded, and the extraction procedure was repeated twice. The solid pellet was air-dried, homogenised, and prepared for analysis with TEM-EDX and DLS. For NEPs where extraction methods yielded enough solid pellet mass, XRD was also performed.

##### Sample Pre-Treatment for CA1–2

A similar extraction method (SUN1–5, LB1) was adopted for CA1–2; the use of organic solvents was eliminated in this case. Briefly, 1 g of sample was introduced in 25 mL of Milli-Q water and vortexed to homogeneity. The suspension was sonicated in a water bath for 30 min at 25 °C and finally centrifuged at 10,000 rpm for 30 min. The organic component of the sample was removed, and the aqueous phase was concentrated by further centrifuging for 30 min at 10,000 rpm, followed by TEM-EDX and DLS analysis.

##### Sample Pre-Treatment for CM1

Two sample pre-treatment methods were performed for the isolation of ENMs from CM1. In the first sample pre-treatment phase, ENMs were isolated from the CM1 matrix using a method previously described (for SUN1-5, LB1). In the second method, a modified method of Bairi et al. [4] for Soxhlet extraction was used. Briefly, 2 g of the sample in a glass fibre thimble (Whatman, Merck, South Africa) was capped with a glass-wool plug that was previously baked at 400 °C. The glass fibre thimble was transferred into the Soxhlet extractor. The 250 mL round-bottom flask was filled with 180 mL of hexane: dichloromethane (1:1) and four boiling chips were added; then, the flask was placed in a heating mantle to initiate the extraction process. The extraction process lasted for 5 h. The organics were extracted into the solvent, while the particulates remained in the thimble. The thimble was air-dried, and the solid material was collected, homogenised, and prepared for TEM-EDX, DLS, and XRD analysis.

##### Sample Pre-Treatment for SAN3, and SK1

SAN3 and SK1 were subjected to two sample pre-treatment methods. For SAN3, the first pre-treatment method was similar to SUN1-5, LB1. In the second pre-treatment method, the ENMs were not isolated from the product matrix; rather, the sample as purchased was transferred into a crucible, frozen at −80 °C for 24 h, and dehydrated using a VirTis^®^ Wizard 2.0 freeze dryer (SP Scientific, Johannesburg, South Africa). Afterwards, the dried sample was homogenised and prepared for analysis.

In the case of SK1, the fabric sample was cut into small pieces and prepared for SEM-EDX analysis in the first pre-treatment method. While in the second method, the modified protocol of Benn and Westerhoff [5] was used. Briefly, the cut pieces were ashed at 550 °C to reduce the bulkiness of the materials. The textile ashes were prepared for analysis. For SEM-EDX analysis, the ashes were directly prepared as highlighted in Section 2.1.1, while for TEM-EDX and DLS, the ashes were firstly dispersed in Milli-Q water and sonicated for 5 min before analysis.

#### 2.1.3. Elemental Quantification of NEPs

The digestion of the samples followed a modified MARS 6 Method Note Compendium [6]. Briefly, 350 mg (SUN1–5, LB1, CA1–2, CM1), 200 mg (SK1), and 1 mL (SAN1–2) of the samples were transferred into the digestion vessels, and 5 mL of HNO_3_ was added (70%, Sigma Aldrich, Johannesburg, South Africa), which was followed by swirling of the vessel and left open for approximately 10 min. After 10 min, 2 mL of H_2_O_2_ (37%, Sigma Aldrich, Johannesburg, South Africa); H_2_O_2_ was replaced with 1 mL of HF (49%, Merck, Johannesburg, South Africa) in samples where Ti was present. The microwave digestion program followed the cosmetics and textiles heating program highlighted in the MARS 6 Method Note Compendium [6] for (SUN1–5, LB1, CA1–2, CM1, SAN1–2) and (SK1), respectively. In cases where the digestion was incomplete, the heating cycle was repeated until the sample was fully solubilised. After sample digestion, HF complexation was performed under conditions stipulated in the CEM Method Note Compendium [6].

A wet digestion method, under low temperature and atmospheric pressure, was used to promote complete digestion of PA1–5 [7]. Briefly, 350 mg of a well-homogenised sample was weighed into a vessel, and 1 mL of HNO_3_ was added. The mixture was heated at 40 °C for 20 min. After cooling, 5 mL of HCl (37%, Merck, South Africa) and 1 mL of HF were added followed by 120 °C heating of the mixture until the sample was fully solubilised (taking approximately 3–4 h). Then, the vessels were transferred to a microwave to perform HF complexation using the conditions stipulated in the MARS 6 Method Note Compendium [6]. All NEPs’ digests were filtered using 0.45 µm (Merck, Johannesburg, South Africa) and prepared for inductively coupled plasma mass spectrometry analysis (ICP-MS, Icap Q, Thermo Fisher Scientific, Waltham, MA, USA), monitoring ^66^Zn, ^48^Ti, ^107^Ag, ^28^Si, ^27^Al, and ^45^Sc (internal standard). The efficiency of all the digestion methods was examined by digesting the standards in bulk form (Zn, Ti, and Ag from Anatech instruments (Johannesburg, South Africa), nTiO_2_ (Tavo-commercial nanocomposite, Merck, Johannesburg, South Africa), nAg (bare and aminated, Nanocomposix, San Diego, CA, USA), nZnO (Z-cote, a commercial nanocomposite, BASF, Johannesburg, South Africa). All the samples and standards were digested and analysed in triplicate; appropriate sample dilutions were performed before ICP-MS analysis.

#### 2.1.4. Data Analysis

Violin plots and statistical analysis were performed using GraphPad Prism 8 version 8.4.3 for Windows (GraphPad Software La Jolla, San Diego, CA, USA). XRD curves were plotted using Microcal™ origin™ version 9.7 (Microcal Software, Inc., Northampton, MA, USA). The Student’s t-test and one-way ANOVA with a Tukey’s HSD post hoc test were applied to test statistical difference at *p*-value of < 0.05.

## 3. Results and Discussion

### 3.1. Characterisation of ENMs in NEPs

The ENMs’ sizes were averaged and reported as width × length, particle size distributions for all NEPs and are presented in Figure S1; the data represent measurements of particles visualised on different locations on the Cu grid. The minimum number of ENMs’ particles measured was set at 50. For CM1 and SK1 (irregularly shaped nTiO_2_ and nAg particles), the number of particles measured was lower than 50 because of the low confidence in identifying and measuring the particles that were associated with NEPs matrix, or the ENMs were distorted. Such is a common analytical limitation experienced in characterising ENMs in complex samples [8].

#### 3.1.1. Sunscreens

**Figure 1:**
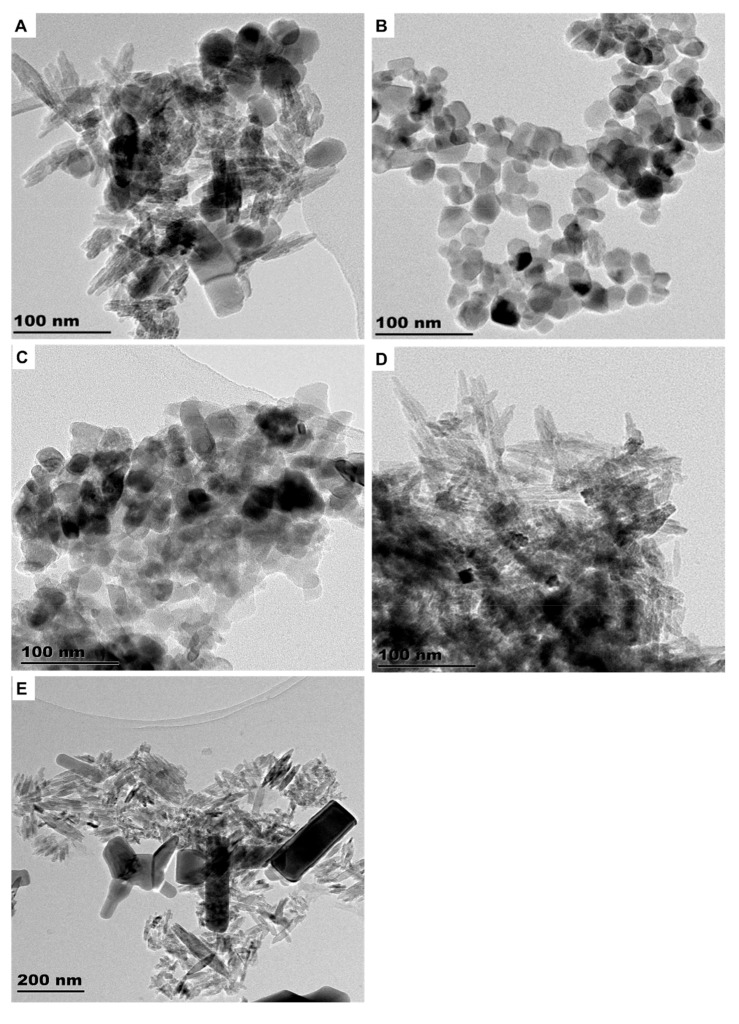
TEM images of engineered nanomaterials (ENMs) found in SUN1 (**A**), SUN2 (**B**), SUN3 (**C**), SUN4 (**D**), and SUN5 (**E**).

**Figure 2:**
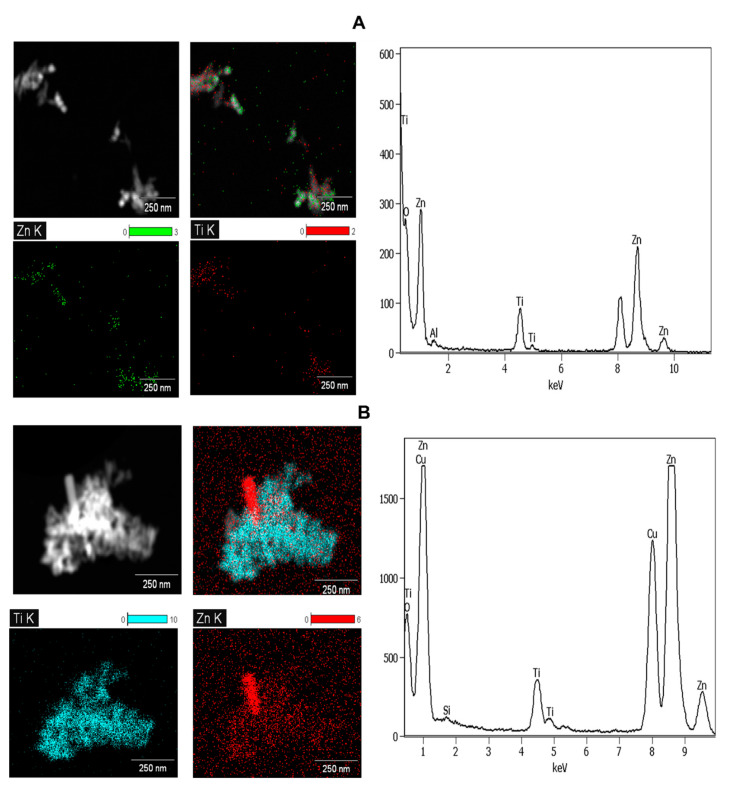
The energy-dispersive X-ray (EDX) elemental mapping for the identification of nTiO_2_ and nZnO detected in SUN1 (**A**) and SUN5 (**B**).

The sunscreens (SUN1–5) were observed to contain ENMs (Figure 1 and Figure S2). The SUN1 contained elongated nTiO_2_ particles with sizes of 14 ± 5 × 62 ± 11 nm and angular nZnO particles with sizes of 35 ± 5 × 38 ± 4 nm. nTiO_2_ and nZnO particles were distinguished through elemental mapping, as depicted in Figure 2. SUN2 and SUN3 contained only angular shaped nTiO_2_ sized 28 ± 4 × 32 ± 5 nm and 20 ± 2 × 27 ± 4 nm respectively; the two SPF50 sunscreens were of the same brand. In SUN4, elongated and angular nTiO_2_ were of sizes 7 ± 2 × 48 ± 11 nm and 14 ± 4 × 17 ± 1 nm, respectively, and they were highly agglomerated compared to SUN1–3. In SUN5 (a suspect NEP), there were elongated nTiO_2_ sized at 9 ± 2 × 72 ± 9 nm, while ZnO particles were larger (129–151 × 254–316 nm) (Figure 1E) and outside the conventional 1–100 nm nano definition; however, it is possible they too had particulates in the nano size.

In all the sunscreens, the ENMs were observed to be still associated with coating agents evident from the EDX spectra in Figure S2. Silicon (Si) (from SiO_2_-based coatings) was observed in SUN2–3 and SUN5. Aluminium (Al; from Al-based coatings) was detected in SUN1, whereas both Si and Al coating were detected in SUN4. The ENMs incorporated in NEPs (sunscreens included) are commonly coated with layers of either Al_2_O_3_, Al(OH)_3_, and Al_2_O_3_ + SiO_2_; these are intended to reduce the photo-reactivity and oxidative stress potential of the ENMs [9,10,11,12]. While the extraction methods may have altered the surface coating agents and agglomeration state, changes in the shape and particle size were not expected. A comparative study of cryo-TEM and TEM observed that only dis-agglomeration occurred between as-purchased and extracted samples [1]. Generally, the ENMs’ size, shape, and elemental composition determined herein were comparable to previous studies [1,3,4,13,14,15,16,17,18].

The ENMs in all the sunscreens were negatively charged (Table S1). The zeta potential of the ENMs ranged between −19 and −52 mV, and since most ENMs were still associated with the ENMs coating agents, it was assumed that their surface charge was not considerably affected by the extraction procedures.

**Figure 3:**
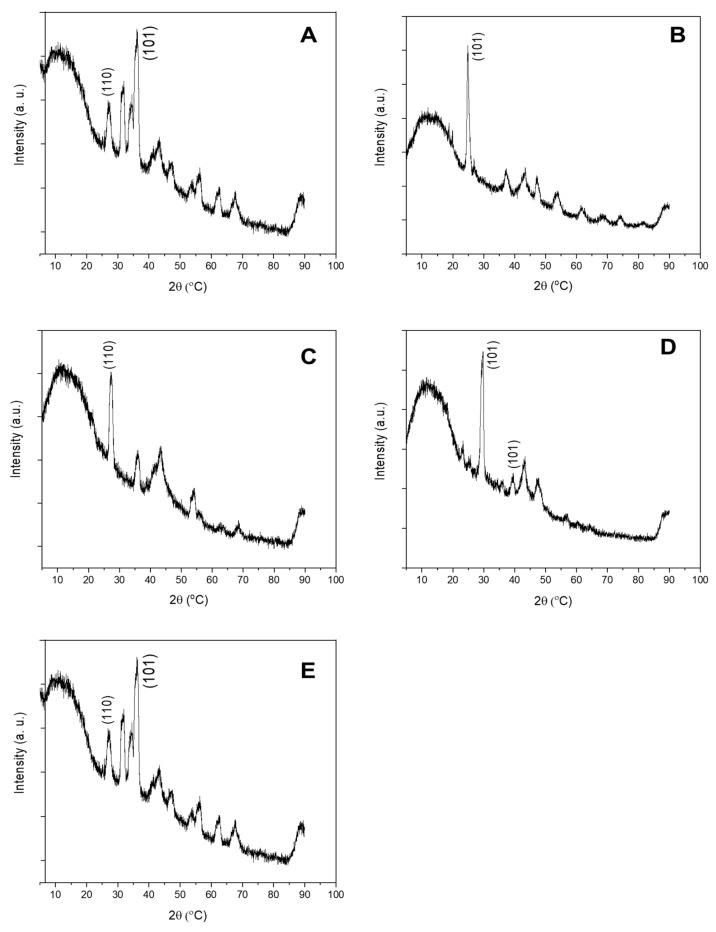
The XRD patterns of SUN1 (**A**), SUN2 (**B**), SUN3 (**C**), SUN4 (**D**), and SUN5 (**E**).

The results of crystal phase determination with XRD are depicted in Figure 3. The phases of nTiO_2_ and nZnO in SUN1 and SUN5 were rutile (ICDD ref code, 01-075-1755) and zincite (ref code, 00-036-1451), respectively. The prominent peaks for TiO_2_ were at 2Ɵ of 27.4, 36.0, 41.2, and 54.3 and respectively corresponding to the (110), (101), (111), and (211) lattice planes. For nZnO, the peaks were at 2Ɵ = 31.7, 34.4, 36.2 47.5, and 56.6, respectively corresponding to (110), (200), (101), (102), and (110). SUN2 contained anatase nTiO_2_ (ICDD ref code, 00-021-1272), with lattice planes of (101) and a corresponding peak at 25.3. SUN3 contained rutile nTiO_2_ (ICDD ref code, 01-078-1508); the prominent peak and the corresponding lattice planes were similar to the nTiO_2_ patterns observed in SUN1 and SUN5. In the case of SUN4, a mixture of rutile (ICDD ref code 01-078-1508) and anatase (ICDD ref code, 00-021-1272) was obtained. The prominent peaks and lattice plane were similar to the respective phases previously reported for the other sunscreens (SUN2 for anatase profile and SUN1 for rutile profile).

The incorporation of rutile [4,13,19], anatase [4,19,20], brookite [21], or a mixture of rutile and anatase [21] in products has been reported previously. Rutile nTiO_2_ is preferred because of its high UV absorption gap (wavelength of 407 vs. 387 nm for anatase) [22] and because of the low photo-reactivity compared to highly reactive anatase that can produce hydroxyl radicals under ultraviolet treatment [23]. A mixture of TiO_2_ forms_,_ as in the case of sample SUN4, appears to be uncommon because only a single case has been reported [21]. So far, the examination of the ZnO crystal structure in sunscreens has been limited to a few reports [4,21]; both studies identified the wurtzite phase of ZnO.

#### 3.1.2. Personal Care Products

**Figure 4:**
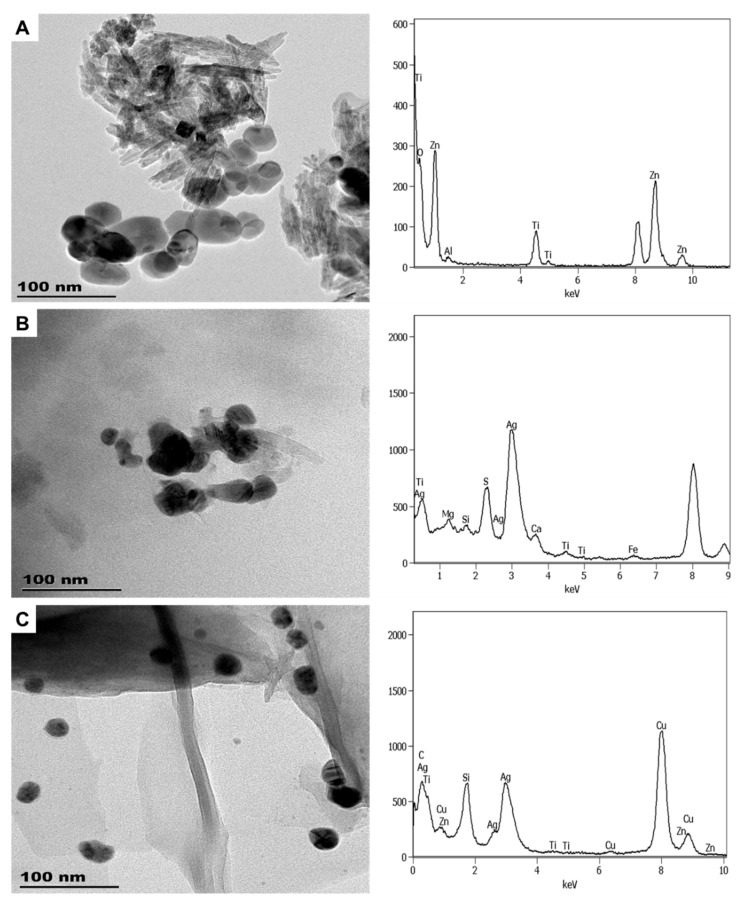
The images and spectra of TEM-EDX analysis of LB1 (**A**), CA1 (**B**), and CA2 (**C**).

**Figure 5:**
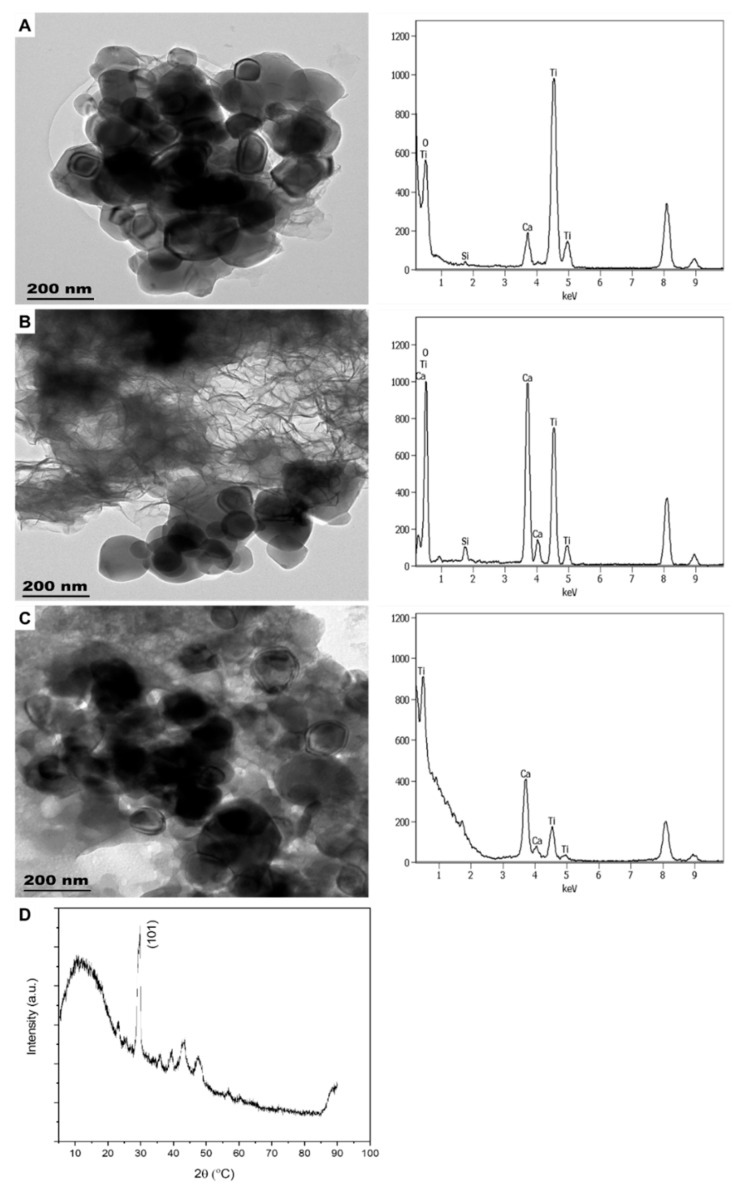
Transmission electron microscopy coupled with energy-dispersive X-ray (TEM-EDX) analysis of ENMs obtained from CM1 after (**A**) sequential ultrasonication, (**B**) Soxhlet extraction, (**C**) diluted nano-enabled products (NEPs), and (**D**) corresponding X-ray powder diffraction (XRD) spectra.

**Figure 6:**
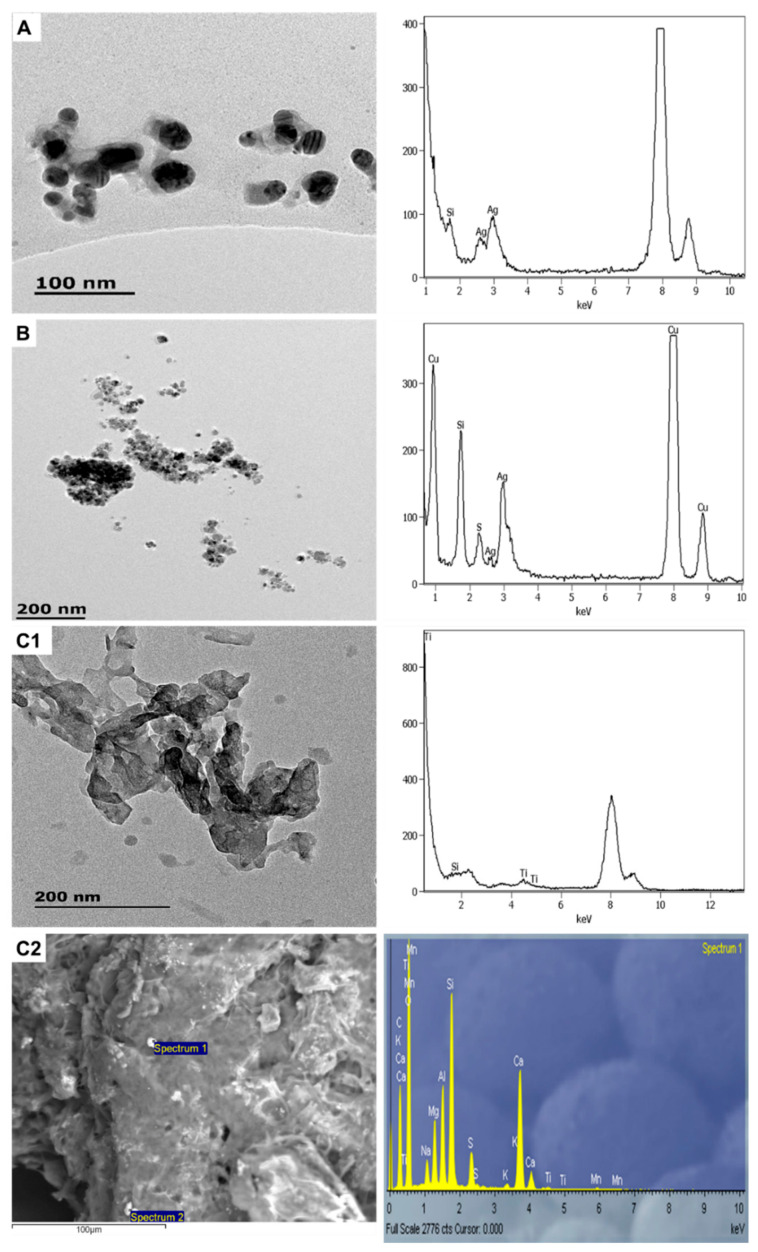
The TEM- and SEM-EDX characterisation of ENMs incorporated in SAN1 (**A**), SAN2 (**B**), and SAN3 (**C1**,**C2**).

Most of the personal care subcategory products were found to be nano-enhanced (Figure 4, Figure 5 and Figure 6), except for SAN3, where no ENMs were detected. As shown in Figure 4, LB1 contained elongated nTiO_2_ (6.5 ± 2 × 52.2 ± 14 nm) and angular nZnO (33 ± 6 × 37 ± 7 nm). XRD analysis of LB1 could not be performed due to the low sample mass obtained after the extraction procedure; this is another practical limitation concerning the examination of ENMs in NEPs, especially toxicity effects assessments [3,24]. Since SUN1 and LB1 were from the same manufacturer and found to contain the same type and comparable size of ENMs; it is most likely that the nTiO_2_ and nZnO used in both products were similar. As in SUN1, the ENMs in LB1 were negatively charged (−19 mV).

The “suspect” products, CA1 and CA2, were found to contain ENMs (Figure 4); thus, they were confirmed as NEPs. CA1 had elongated nTiO_2_ sized 8 ± 2 × 53 ± 18 nm and near-spherical nAg sized 27.5 ± 7 nm. CA2 had near-spherical nAg particles with a size range of 21.7 ± 6 nm. Although CA1 and CA2 were from different manufacturers, their nAg sizes were relatively similar. The zeta potentials of the ENMs in CA1 and CA2 were closely related at −34 and −36 mV, respectively. Similar to LB1, XRD analysis for CA1–2 could not be undertaken because of the low sample mass obtained after the extraction procedure.

According to the manufacturer, CM1 contained nSiO_2_; however, only the bulk form was detected (Figure 5). Furthermore, even after employing different extraction procedures, SiO_2_ in the nano-form was not observed, but instead, nTiO_2_ was detected. The nTiO_2_ was angular in shape and had a size range of 32–151× 62–168 nm; sizes obtained from different sample pre-treatments were comparable, indicating that none of the methods employed distorted the particle size. The nTiO_2_ was negatively charged (−18 mV) and of anatase form (ICDD ref code, 00-021-1272); the XRD patterns were similar to those of SUN2.

The sanitisers (SAN1–2) contained near-spherical nAg (Figure 6). SAN1 contained two distinct particle sizes of 22 ± 7 and 37 ± 4 nm; the larger counterparts appeared to be due to an agglomeration effect. The nAg contained in SAN2 was 20 ± 4 nm in size. The ENMs’ surface charge in SAN1 and SAN2 was comparable at −22 mV and −23 mV. XRD analysis was not undertaken on SAN1–2 as they were in liquid form. SAN1 and SAN2 were both not labelled to be nano-enhanced, but analysis undertaken herein confirmed them to be NEPs. In SAN3, no ENMs were found even after exploring different sample preparation techniques (Figure 6C1,C2); thus, no further characterisation was undertaken.

#### 3.1.3. Socks

**Figure 7:**
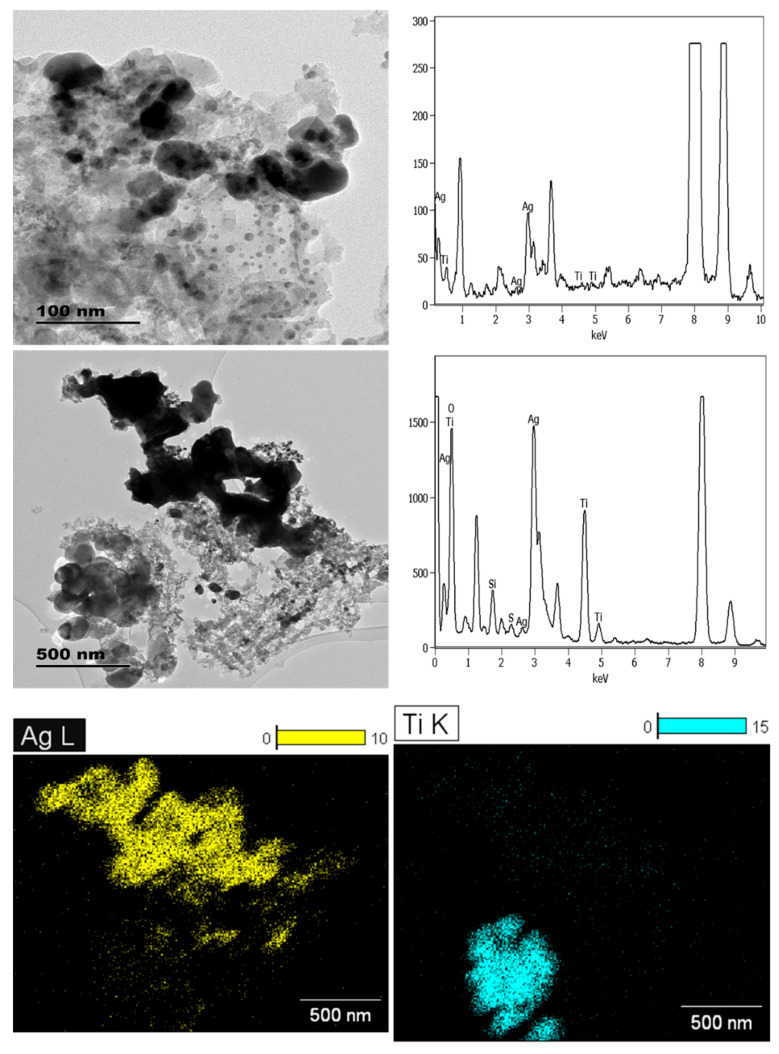
TEM-EDX analysis of ashed SK1 and elemental mapping showing the presence of nAg (yellow) and nTiO_2_ (blue).

For clothing NEPs, SK1 contained nAg and nTiO_2_, where the manufacturer had only declared the incorporation of nAgCl. The nAg was near-spherical and irregular, whereas nTiO_2_ was angular (32–203 × 48–135 nm). The near-spherical nAg particles were sized 18 ± 5 nm, while irregularly shaped counterparts consisted of two size sets of 36 ± 9 × 34 ± 13 nm and 85 ± 21 × 65 ± 33 nm. Although ENMs’ size and shape were established, in some cases, the ashing procedure altered the particle shape of nAg in/on the sock (Figure 7). The ENMs surface charge of ashed material was −13 mV; it is most likely that the charge of the ENMs was affected by the ashing procedure.

The irregularly shaped nAg appeared to be ashing-induced agglomeration. Though ashing interfered with the ENMs shape in SK1, the procedure was deemed necessary to remove the bulkiness of the sock fabric material and thus revealing the ENMs. EDX elemental mapping distinguished the nTiO_2_ from nAg in SK1 (Figure 7). To date, the characterisation of ENMs in fabrics has mainly been on laboratory-developed products [25,26,27,28,29], as compared to commercial textiles in which 100–500 nm nearly spherical nAg and irregular shaped (<100 nm) have been reported [5,30].

**Figure 8:**
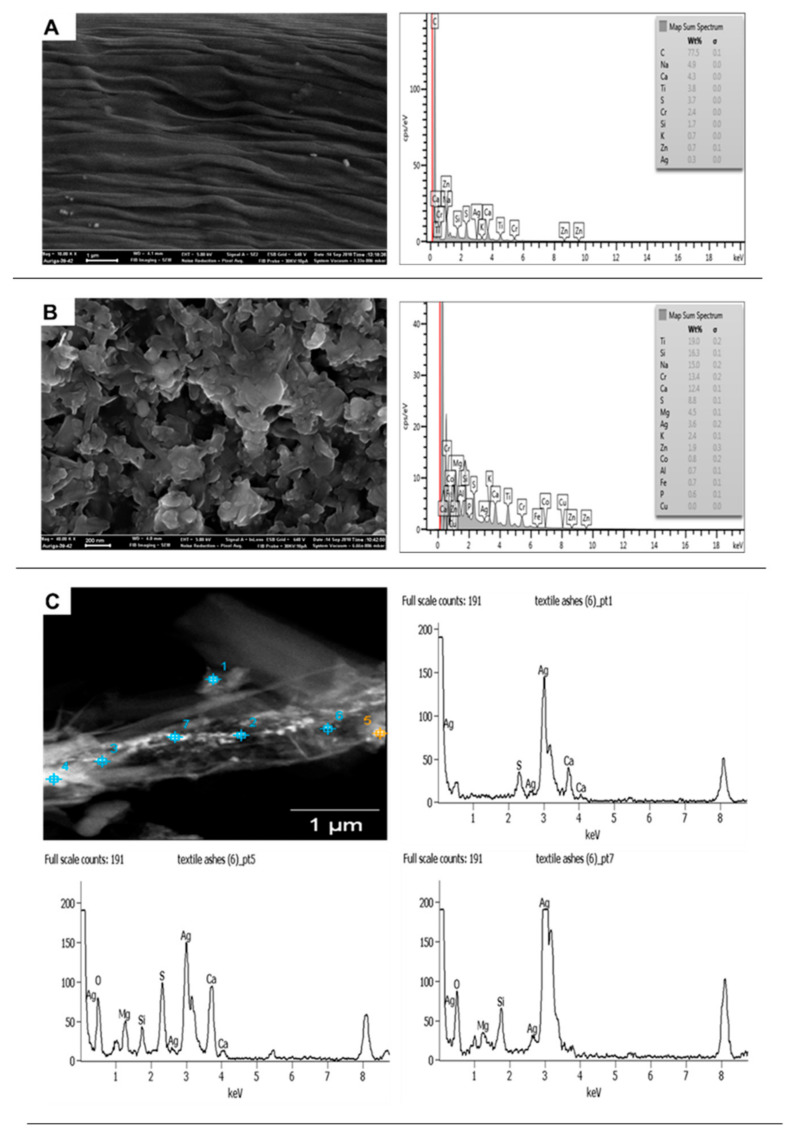
Scanning electron microscopy coupled to energy-dispersive X-ray (SEM-EDX) images of non-ashed (**A**) and ashed (**B**) SK1 and TEM-EDX of SK1 showing nAg in a straight line within the fibre (**C**).

SEM-EDX characterisation data for SK1 was generally inconclusive (Figure 8A,B). The lack of detection of ENMs by SEM-EDX (a surface characterisation technique) suggested that the ENMs were inside the NEPs matrix and not surface coated. This possibility was further supported by TEM-EDX analysis (Figure 8C), whereby the ENMs (especially nAg) were seen to be deposited in a straight line inside the textile fibre. The location of ENMs in NEPs plays a significant role in the release potential of ENMs; for example, ENMs that are surface coated exhibit high release potential as compared to ENMs suspended in solid/matrix [19]. Similar to other personal care products (excluding CM1), the sample mass obtained after SK1 ashing was not sufficient to perform XRD analysis.

#### 3.1.4. Paints

**Figure 9:**
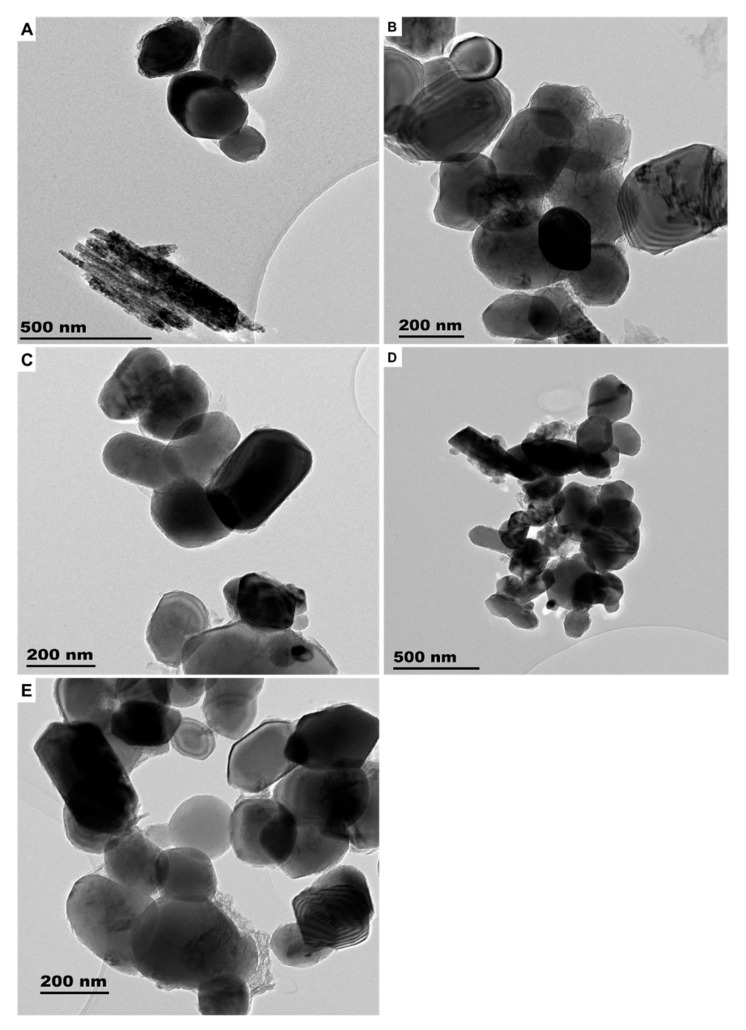
TEM images obtained from the analysis of PA1 (**A**), PA2 (**B**), PA3 (**C**), PA4 (**D**), and PA5 (**E**).

**Figure 10:**
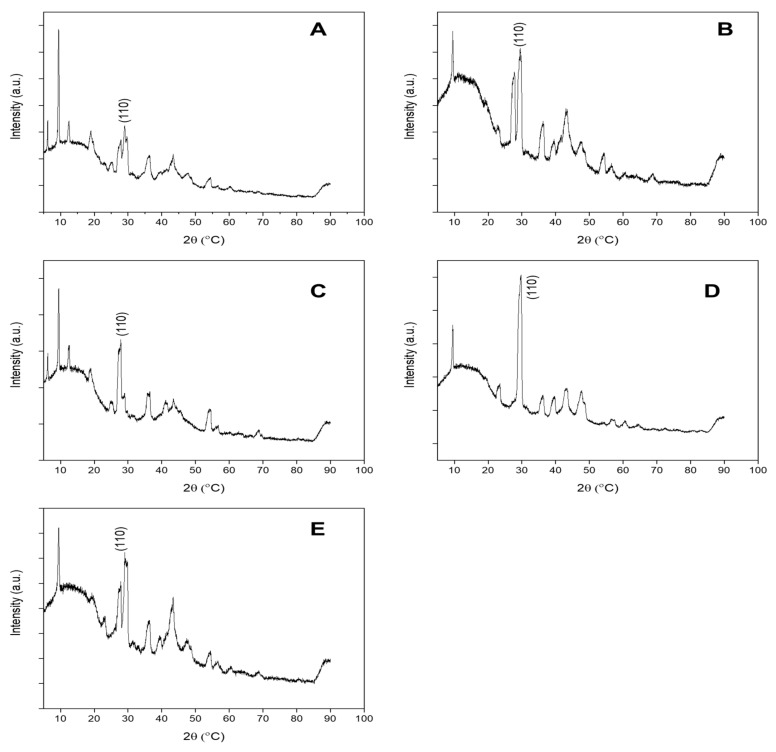
The XRD patterns of PA1 (**A**), PA2 (**B**), PA3 (**C**), PA4 (**D**), and PA5 (**E**).

Angular particles of nTiO_2_ with varying sizes were detected in all paint “suspects” (PA1–5) (Figure 9 and Figure S3). The nTiO_2_ were measured to be 200 ± 56 × 253 ± 100 nm (PA1), 199 ± 47 × 251 ± 84 nm (PA2), 197 ± 49 × 239 ± 62 nm (PA3), 168 ± 53 × 239 ± 60 nm (PA4), and 175 ± 41 × 178 ± 48 nm (PA5). Fe-based ENMs with an elongated rod structure of 40 ± 12 × 297 ± 139 nm size were also observed in PA1. Angular nTiO_2_ ranging 90–300 nm has previously been reported in paints [31,32]. The ENMs in paints were all negatively charged and ranged between −12 and −20 mV (Table S1). The nTiO_2_ incorporated in PA1–PA5 was all in the rutile phase (Figure 10), with ICDD ref codes 00-021-1276 (PA1), 01-076-0649 (PA2), 01-072-1148 (PA3, PA4), and 01-073-2224 (PA5). The prominent peaks and the corresponding lattice plane were observed at 2Ɵ = 27.4 (110), 36.0 (101), and 54.3 (211). The use of anatase nTiO_2_ in paints has previously been reported [32].

### 3.2. Elemental Quantification of ENMs in NEPs

The total elemental concentrations of the labelled and suspect NEPs are reported in Table 2. The analysis techniques were satisfactory, as the recoveries of the standards highlighted in Section 2.1.3 were in the ranges of (75–107%) Ti, (72–97%) Ag, and (74–98%) Zn. The elements of ENMs that were not declared or suspected, but observed during sample characterisation, were also quantified and reported in Table 2. The ENMs quantities, irrespective of ENMs type in paints products, are seldomly reported. From the commercial paints incorporated with bulk TiO_2_, Ti concentrations ranged between 0.00044 and 2.8% (*w*/*w*) [7,41].

The amounts determined in SUN1–5 were in agreement with a previously reported total Ti concentration of 0.34–13.1% (*w*/*w*) [1,19,33,34,35] and total Zn concentration of 2–20% (*w*/*w*) [14]. The amounts found in Ag-based personal care products SAN1–2 and CA1–2 were found to be lower than those declared by manufacturers; CA2 was an exception, where the determined concentration of 14 mg/L was closer to the 18 mg/L declared by the manufacturer.

The difference between the determined and declared quantity is not uncommon; for example, Cascio et al. [36] and Wasukan et al. [37] found that in some products, the listed and determined amounts vary by as much as ten-fold, but in some products, the determined and declared quantities were relatively similar. Sometimes, there can be differences between product batches from the same manufacturer [38]. Although the amounts determined for nAg-based NEPs were commonly found to be lower than the declared, the amounts often agreed with those reported in the literature. For example, the quantity of total Ag in SK1 was comparable (0.00015–0.29% (*w*/*w*)) to previous studies investigating nAg in nano-enabled socks; the incorporation of Ag content ranging from 0.0001 to > 1% (*w*/*w*) has been considered normal in commercial textiles [30].

The quantification of Ti in commercial textile NEPs is rare; in the few available reports, the total Ti was reported to be at 0.00026–0.145% (*w*/*w*) [40]. Windler et al. [40] reported total Ti at 0.68–0.71% (*w*/*w*) in commercial textiles that were not nano labelled by the supplier company but were confirmed to be nTiO_2_. The same study further reported total Ti amounts in the range of 0.30–0.85% (*w*/*w*) in textile products that had no nano labelling. The ENMs quantities, irrespective of ENMs type in paints products, are seldomly reported. From the commercial paints incorporated with bulk TiO_2_, Ti concentrations ranged between 0.00044 and 2.8% (*w*/*w*) [7,41].

Thus far, the amount of ENMs added in various commercial NEPs has mostly been determined in the products as a whole using conventional spectrometric techniques such as inductively coupled plasma (ICP) coupled to different detectors (X) [1,7,14,15,19,30,33,34,36,37,39,40,42,43]. Conventional spectrometric techniques analysis requires prior sample pre-treatment such as acid digestion and an analysis method (elemental analysis) that quantifies both the bulk and nano particulates; thus, the accurate determination of ENMs amounts is not achieved. The quantification of ENMs added in NEPs specific to the particle are scarce. In rare cases, single-particle inductively coupled plasma mass spectrometry (spICP-MS) quantified the particle concentration of nTiO_2_ at 1.9 × 10^2^–4.8 × 10^5^ parts/mL [15,42,43] and nZnO at 1.4 × 10^3^–1.0 × 10^4^ particle/mL [43] in sunscreens and 1.0 × 10^4^–3.9 × 10^5^ particles/mL in personal care products (i.e., shampoos, facial cream, toothpaste, lip balm, and day cream) [42]. The determination of particle concentration of ENMs added in NEPs is emerging [39]; the growing application of spICP-MS and reporting of ENMs particle concentration in complex environmental media has been reported [44,45].

Overall, the amounts of ENMs determined using spICP-MS (particle/volume) and conventional spectroscopic techniques (mass/volume) differ; the difference is not consistent, and different studies reported an overestimation or underestimation, and no difference in the amounts of ENMs [15,39,43,44,46]. The difference is due to various factors, one being that the conventional spectroscopic techniques determine the total concentration of the target analyte (bulk, ENMs, and ions), while spICP-MS only determines the ENMs particle concentration [47,48]. Sample preparation, dilution, and evaporation in spICP-MS and acid digestion in ICP-X techniques also possibly contribute toward the different amounts determined by the two techniques [39].

While both techniques provide the amount of ENMs added in NEPs, quantification using such methods is associated with drawbacks. For example, the sample preparation time is longer with conventional spectroscopic techniques compared to spICP-MS, and the concentration specific to the ENMs is lost. spICP-MS techniques suffer from isobaric interferences; correcting the isobaric interferences often leads to decreased sensitivity, thus increasing the size detection limit [39]. Furthermore, it is challenging to obtain consistent/reproducible measurements of size, size distribution, and the number of particle concentration using spICP-MS [49]. Nonetheless, spICP-MS is a promising technique that can rapidly provide comprehensive physico-chemical properties data of ENMs (i.e., particle concentration, size distribution, apparent core density, and state of aggregation) [47,50,51].

## 4. Concluding Remarks

In pursuit of advancing knowledge with regard to the environmental implications of nanotechnology, it is essential that the estimation of ENMs exposure potential be highly relevant to the ENMs lifecycle. The current study sought to expand knowledge about the physico-chemical characteristics of ENMs in NEPs as potential sources of nanopollution; the study was exclusive to a sample of NEPs that exhibit high likelihood to nanopollute water resources.

The complementary analytical methods applied in this study were largely successful in establishing the physico-chemical properties of ENMs in NEPs. Seventeen (94%) products examined herein contained ENMs and were thus confirmed to be NEPs. The “suspects” products, namely, SUN5, CA1–2, SAN1–2, and PA1–5 were determined to contain ENMs and were thus confirmed as NEPs. Due to the limited regulatory requirement for manufacturers to nano-declare, consequentially, the modelling estimations of environmental exposure solely based on nano-declared products may be underestimated, thus resulting in lower risk estimation. It is on such basis that efforts to better estimate the extent of nanopollution can be enhanced by introducing mandatory declaration of NEPs by manufacturers (reliable country NEPs inventory) solely to establish the baseline of nanopollution potential.

Generally, the ENMs in NEPs widely differed (in type, size, shape, and composition) but were all negatively charged. Of the 17 NEPs, 9 (52%), 3 (18%), 3 (18%), and 2 (12%) were incorporated with nTiO_2_, nAg, binary nZnO + nTiO_2_, and nTiO_2_ + nAg, respectively. Hence, ENMs’ environmental emissions (i.e., nanopollution) from NEPs can be generally expected to be in that order, provided that market penetration and product life cycle dynamics are not factored.

The characterisation of liquid suspended ENMs (SAN1–2) was relatively easier compared to those in semi-liquid NEPs (SUN1–5, CA1–2, CM1, and PA1–5) and surface-bound (SK1); therefore, more efforts are needed to develop reliable and even standardised characterisation methods especially for the former. The findings further strengthened the applicability of the “ENMs fixation parameter” for low tier estimation of potential for nanopollution.

Overall, the study demonstrated that some existing conventional techniques are capable to a large extent of characterising ENMs in NEPs, as long as the sample techniques effectively isolate the ENMs from the other product components. Inappropriate and ineffective techniques for ENMs isolation can introduce artefacts and cause misinterpretation of the results. We raise that the determination of the concentration of the ENMs solely based on the total elemental concentration remains a global weakness in studies of this nature, arising from the inadequacy of current techniques and limited accessibility/awareness of some of the latest options. On that basis, we recommend heightened efforts to test and refine some of the relatively new techniques, for instance, the spICP-MS and Nanoparticle Tracking Analysis for more accurate characterisation of the concentration parameter, coupled with effective techniques for ENMs isolation.

## Figures and Tables

**Table 1:** The sample of nano-enabled products (NEPs) examined. ^a^ and ^b^ respectively indicate suspect products and engineered nanomaterials (ENMs).

Sample	Product Type	ENMs Type (Labelled/Suspected ^b^)	ENMs Location	Ingredient Description/NEPs Functionality Declaration
SUN1	Sunscreen	TiO_2_ + ZnO	Suspended in liquid	Reflective sun protection (UVA/ UVB)
SUN2	Sunscreen	TiO_2_	Suspended in liquid	UVA/UVB immediate protection
SUN3	Sunscreen	TiO_2_	Suspended in liquid	UVA/UVB immediate protection
SUN4	Sunscreen	TiO_2_	Suspended in liquid	UVA/UVB immediate protection
SUN5 ^a^	Sunscreen	TiO_2_ + ZnO ^b^	Suspended in liquid	Advanced sun protection
LB1	Lip balm	TiO_2_ + ZnO	Suspended in liquid	Reflective sun protection (UVA/ UVB)
CA1 ^a^	Body cream	Ag ^b^	Suspended in liquid	Ionic colloidal silver, antibacterial
CA2 ^a^	Body cream	Ag ^b^	Suspended in liquid	Ionic colloidal silver, anti-inflammatory
CM1	Cream activator	SiO_2_	Suspended in liquid	Nano
SAN1 ^a^	Sanitiser	Ag ^b^	Suspended in liquid	Ionic colloidal silver, antibacterial
SAN2 ^a^	Sanitiser	Ag ^b^	Suspended in liquid	Colloidal silver
SAN3 ^a^	Sanitiser	Ag ^b^	Suspended in liquid	Antibacterial
PA1 ^a^	Paint	Ag, TiO_2_, ZnO, SiO_2_^b^	Suspended in liquid	Nanotechnology
PA2 ^a^	Paint	Ag, TiO_2_, ZnO, SiO_2_^b^	Suspended in liquid	Nanotechnology
PA3 ^a^	Paint	Ag, TiO_2_, ZnO, SiO_2_^b^	Suspended in liquid	Antibacterial
PA4 ^a^	Paint	Ag, TiO_2_, ZnO, SiO_2_^b^	Suspended in liquid	New technology
PA5 ^a^	Paint	Ag, TiO_2_, ZnO, SiO_2_^b^	Suspended in liquid	Advanced technology
SK1	Socks	AgCl	Surface bound	Nano, antibacterial

**Table 2:** The total elemental concentration of the ENMs found in NEPs.

Sample	Target Analyte	Claimed Concentration (%)	Concentration (%)
SUN1	Zn	not listed	4.31 ± 0.86 ^b^
	Ti	not listed	1.72 ± 0.41 ^b^
SUN2	Ti	not listed	0.945 ± 0.06 ^b^
SUN3	Ti	not listed	1.62 ± 0.08 ^b^
SUN4	Ti	not listed	2.10 ± 0.06 ^b^
SUN5	Zn	not listed	6.84 ± 0.56 ^b^
	Ti	not listed	2.60 ± 0.32 ^b^
LB1	Zn	not listed	3.40 ± 0.04 ^b^
	Ti	not listed	1.41 ± 0.35 ^b^
CA1	Ag	1.80 × 10^−3^ ^a^	8.25 × 10^−4^ ± 3.54 × 10^−5^ ^b^
	Ti	not listed	2.31 × 10^−4^ ± 8.8 × 10^−6^ ^b^
CA2	Ag	1.80 × 10^−3^ ^a^	1.46x10^−3^ ± 1.7 × 10^−5^ ^b^
CM1	Ti	Not listed	0.949 ± 0.04 ^b^
SAN1	Ag	1.80 × 10^−3^ ^a^	11.3 × 10^−3^ ± 3.18 × 10^−5^ ^c^
SAN2	Ag	1.80 × 10^−3^ ^a^	8.13 × 10^−4^ ± 3.0 × 10^−5^ ^c^
PA1	Ti	not listed	2.09 ± 0.24 ^b^
PA2	Ti	not listed	2.79 ± 0.55 ^b^
PA3	Ti	not listed	2.76 ± 0.43 ^b^
PA4	Ti	not listed	0.22 ± 0.02 ^b^
PA5	Ti	not listed	1.67 ± 0.23 ^b^
SK1	Ag	2.00 ^b^	0.181 ± 0.004 ^b^
	Ti	not listed	1.31 ± 0.07 ^b^

^a^ = (*w*/*v*), ^b^ = (*w*/*w*), ^c^ = (*v*/*v*).

## Data Availability

The data presented is available on request from the corresponding author.

## Supplementary Materials

The following are available online, Figure S1: A Violin plot showing the overall particle size distribution of ENMs characterised in NEPs. Figure S2: EDX of ENMs found in SUN1 (A), SUN2 (B), SUN3 (C), SUN4 (D), and SUN5 (E). Figure S3: EDX spectra’s obtained from the analysis of PA1 (A), PA2 (B), PA3 (C), PA4 (D), and PA5 (E)., Table S1: Zeta potential of the ENMs (extracts) incorporated in NEPs.

## Sample Availability

Samples of the compounds are available from the authors.
