# Supplementary material for: Characterisation of Engineered Nanomaterials in Nano-Enabled Products Exhibiting Priority Environmental Exposure
Source: Molecules. 2021 Mar 4;26(5):1370. doi: 10.3390/molecules26051370 (PMC7961725; doi:10.3390/molecules26051370)
Supplement: Supplementary file 1 [file molecules-26-01370-s001.pdf]

Characterisation of engineered nanomaterials in nano-enabled products exhibiting priority environmental exposure

Lehutso RF[1,2], Tancu Y[1], Maity A[2,3], Thwala M[1,4] MThwala@csir.co.za

[1]Water Centre, Council for Scientific and Industrial Research, Pretoria, South Africa

[2]Department of Chemical Sciences, University of Johannesburg, Johannesburg, South Africa

[3]DST/CSIR, Centre for Nanostructure and Advanced Materials (CeNAM), Council for Scientific and Industrial Research, Pretoria 0001, South Africa

[4]Department of Environmental Health, Nelson Mandela University, Port Elizabeth, South Africa

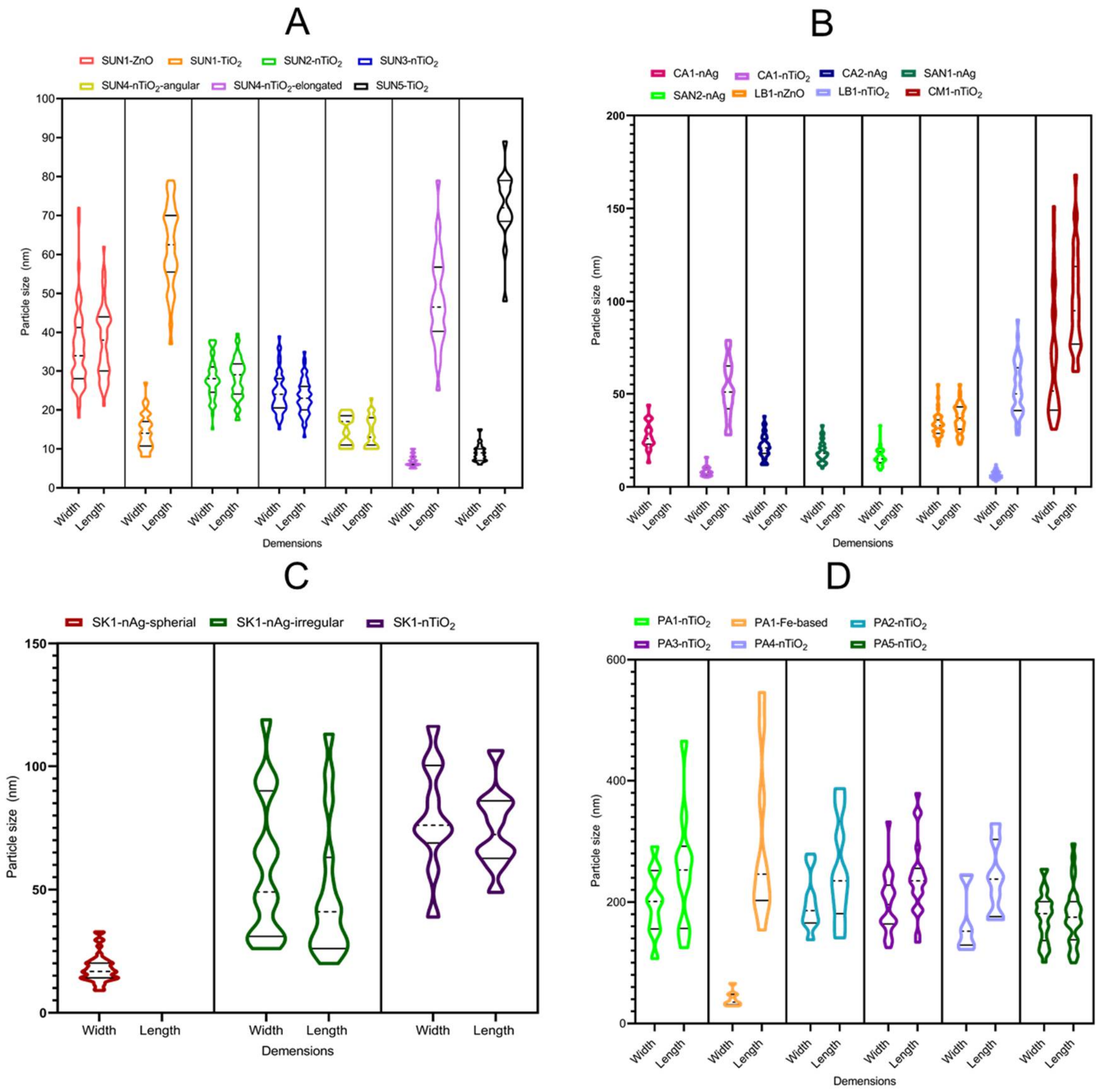

Figure S1: A Violin plot showing the overall particle size distribution of ENMs characterised in NEPs sub-categorised in sunscreens (A), personal care products (B), clothing (C), and paints (D). Upper and lower quartiles are highlighted by a solid line, while the dotted line indicates the median. The denser the violin shape the higher the number of the particle size in that region.

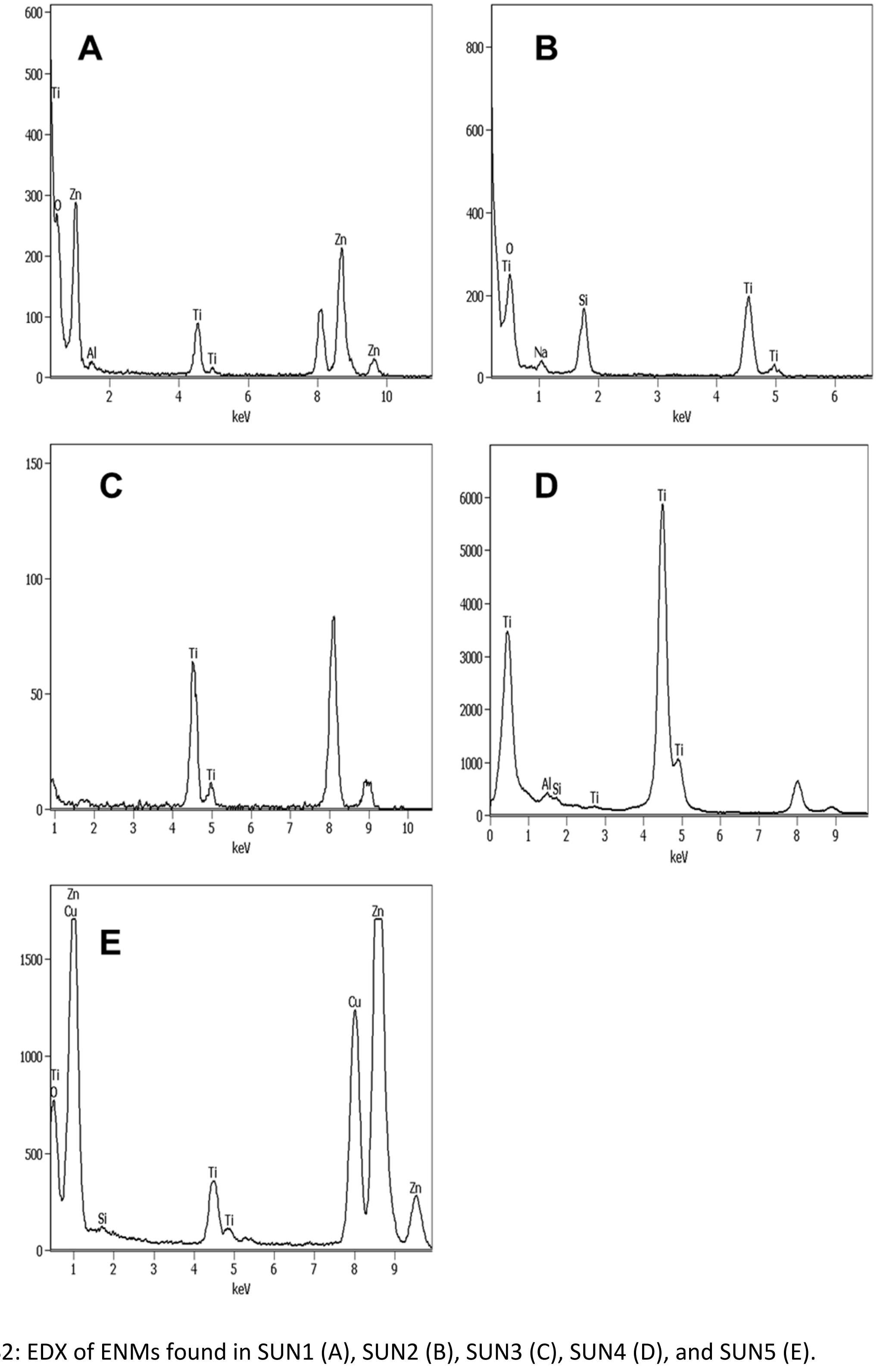

Figure S2: EDX of ENMs found in SUN1 (A), SUN2 (B), SUN3 (C), SUN4 (D), and SUN5 (E).

Table S1: Zeta potential of the ENMs (extracts) incorporated in NEPs

Product	Zeta potential (mV)
SUN1	-28.7±4.9
SUN2	-37.9±8.0
SUN3	-52.2±9.43
SUN4	-48±5.8
SUN5	-19.2±1.0
LB1	-19.2±3.17
CA1	-33.5±0.66
CA2	-36.3±1.5
CM1	-18.1±4.1
SAN1	-21.6±0.85
SAN2	-23.5±1.1
PA1	-14.7±2.2
PA2	-16.3±0.88
PA3	-18.8±0.79
PA4	-13±2.7
PA5	-15.8±0.4
SK1	-12.9±1.8

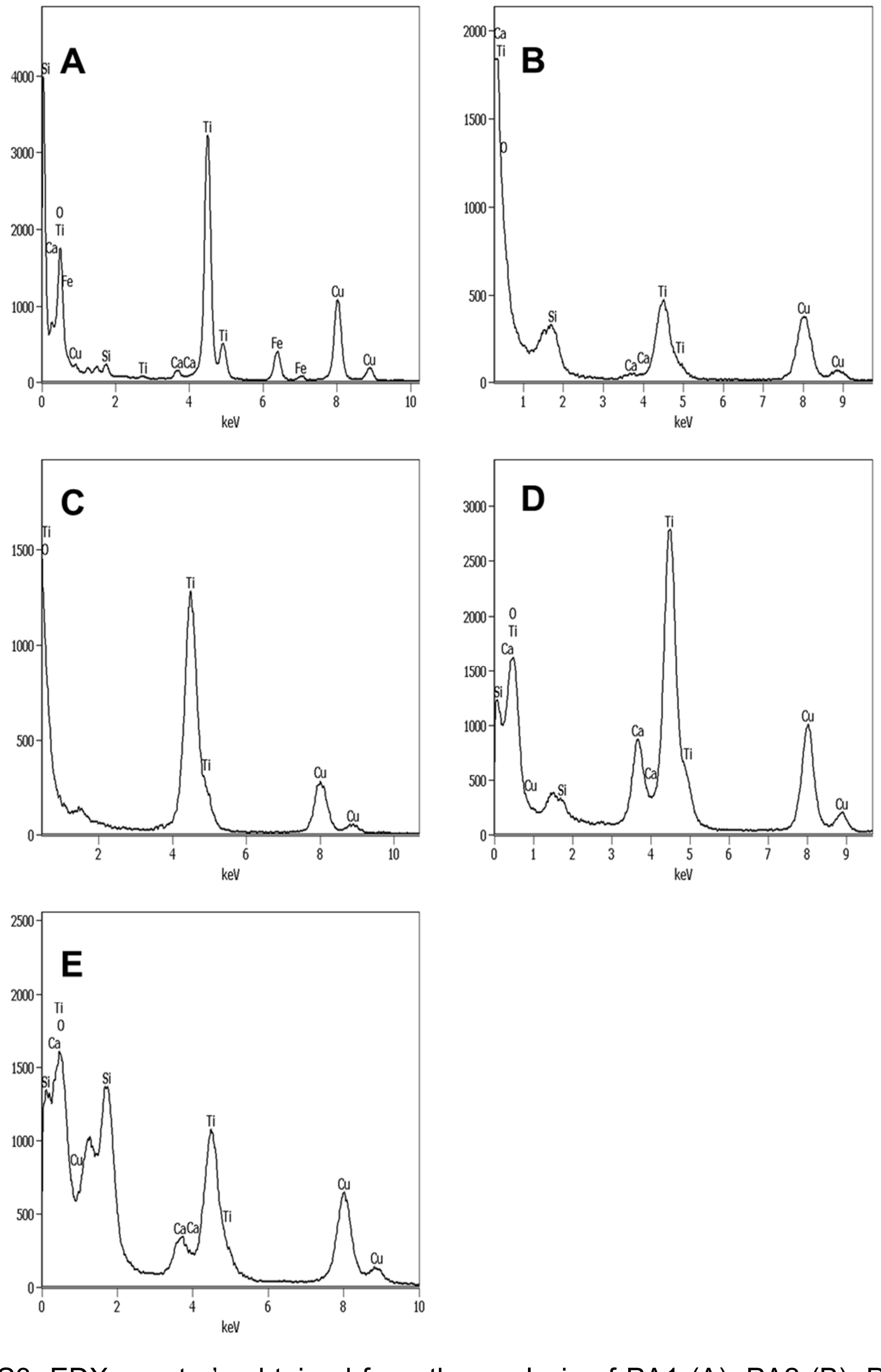

Figure S3: EDX spectra's obtained from the analysis of PA1 (A), PA2 (B), PA3 (C), PA4 (D), and PA5 (E).
